# From MAiD Referral to Targeted Therapy Success: A Case of *BRAF*-Mutated Anaplastic Thyroid Cancer

**DOI:** 10.3390/reports9010010

**Published:** 2025-12-28

**Authors:** Brett Stubbert, Paul Stewart, Eric Winquist, Matthew Cecchini, Claire Browne

**Affiliations:** 1 Schulich School of Medicine & Dentistry, Western University, London, ON N6A 5C1, Canadaeric.winquist@lhsc.on.ca (E.W.);; 2 Division of Medical Oncology, Department of Oncology, London Health Sciences Centre, London, ON N6A 5W9, Canada; 3 Department of Pathology, London Health Sciences Centre, London, ON N6A 5W9, Canada; 4 Department of Medical Oncology, Arthur Child Comprehensive Cancer Centre, Calgary, AB T2N 5G2, Canada

**Keywords:** anaplastic thyroid cancer, BRAF V600E, immunohistochemistry, next-generation sequencing, single-payer medication funding

## Abstract

**Background and Clinical Significance**: Anaplastic thyroid cancer (ATC) is a rare and aggressive malignancy with a poor prognosis, where median survival typically ranges from 4 to 10 months. Advances in genetic profiling, particularly the identification of *BRAF* mutations, offer new opportunities for targeted therapy. **Case Presentation**: This case report details the journey of a woman in her late 50s diagnosed with symptomatic ATC. Initial immunohistochemistry (IHC) testing for *BRAF* mutations returned negative results, leaving the patient with limited treatment options and prompting her to pursue medical assistance in dying (MAiD). However, next-generation sequencing (NGS) confirmed a *^V600E^BRAF* mutation, and a basis for targeted therapy. The patient began treatment with dabrafenib-trametinib, followed by pembrolizumab as second-line therapy, ultimately extending her life by nearly seven months. **Conclusions**: This case underscores the importance of rapid and comprehensive diagnostic approaches, particularly the higher sensitivity of NGS over IHC for detecting *BRAF* mutations. The complexities of accessing newer therapies in Canada’s single-payer healthcare system are also emphasized. The utilization of newer rapid diagnostic technologies can have a direct impact on directing treatment for ATC and other aggressive malignancies.

## 1. Introduction and Clinical Significance

Anaplastic thyroid cancer (ATC) is the most aggressive and lethal thyroid malignancy, representing less than 2% of all thyroid cancers but accounting for 19–39% of thyroid cancer deaths [1]. ATC is characterized by rapid growth, local invasion, and early metastasis, often leading to a poor prognosis with a median survival of only 4 to 10 months despite aggressive treatment [2]. The presence of *BRAF* gene mutations, particularly the *^V600E^BRAF* mutation, has been identified in 25 to 40 percent of ATC cases, offering potential therapeutic targets for personalized treatment strategies [3,4,5].

Traditional treatment options for ATC include surgery, radiation therapy, and chemotherapy [6]. However, due to the aggressive nature of ATC, these approaches often provide limited efficacy. The introduction of targeted therapies, such as dabrafenib and trametinib for *BRAF*-mutant ATC, has brought new hope in managing this challenging disease. Dabrafenib, a *BRAF* inhibitor, and trametinib, a *MEK* inhibitor, work synergistically to inhibit the Mitogen-Activated Protein Kinase/Extracellular signal-Regulated Kinase (*MAPK/ERK*) pathway, which is crucial for the growth and survival of *BRAF*-mutant cancer cells [7].

Recent clinical studies have demonstrated significant clinical responses in patients with *BRAF*-mutant ATC treated with dabrafenib and trametinib. For instance, Subbiah et al. reported a 69% overall response rate in patients treated with this combination, with durable responses observed in a majority of cases [7]. Additionally, the programmed death ligand 1 (PD-L1) inhibitor pembrolizumab has shown potential as an immunotherapy option, especially in tumours expressing high levels of PD-L1 [8].

Here, we present the case of a patient diagnosed with *BRAF*-mutant anaplastic thyroid cancer with an unusual diagnostic course highlighting nuances in molecular testing.

## 2. Case Presentation

**Figure 1:**
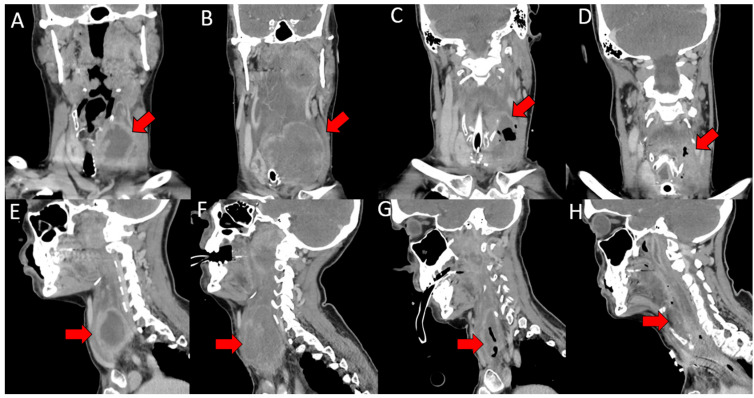
Computed tomography (CT) neck images of the patient demonstrating the size changes in the primary tumour; the tumour is indicated with the red arrow in all images. Images (**A**–**D**) are coronal plane images and images (**E**–**H**) are sagittal plane images. (**A**,**E**): Earliest CT imaging, at initial presentation, showing a left-sided neck tumour beginning to displace the trachea, measuring 6.0 × 4.4 × 4.7 cm. (**B**,**F**): CT imaging from 2 months post-presentation, with the patient intubated in intensive care, showing the primary tumour enlarged to 8.1 × 7.8 × 5.0 cm. (**C**,**G**): CT imaging from 2.5 months post-presentation after 9 days of dabrafenib-trametinib administration, displaying a shrinkage of tumour to 7.1 × 6.3 × 3.8 cm. (**D**,**H**): CT imaging from 5 months post-presentation, after 3 months of dabrafenib-trametinib administration, tumour approximately 3.2 × 3.5 × 4.3 cm.

A 58-year-old female presented with a rapidly enlarging symptomatic thyroid mass. She had been previously well with no past medical history and no risk factors for thyroid malignancy. Initial computed tomography (CT) neck imaging revealed a 6.0 × 4.4 cm heterogeneous mass replacing the left thyroid lobe, exerting significant mass effect on the trachea and esophagus, with likely invasion into the left lateral and anterior esophageal muscularis (Figure 1A,E). Necrotic lymph nodes and ipsilateral cervical adenopathy were noted. The patient reported progressive dysphagia, severe enough that she could only swallow a teaspoon of water and had lost 20 pounds over four weeks. Due to rapidly progressive symptoms, ATC was clinically suspected. Initial fine needle aspiration (FNA) of the patient’s thyroid revealed nondiagnostic findings suspicious for carcinoma. The patient then underwent additional open thyroid biopsy, where three 4 mm punch biopsies were taken, again yielding nondiagnostic findings.

The patient was admitted to hospital one month after presentation due to escalating symptoms of progressive dysphagia, decreased vocal cord mobility, and paralysis of her left vocal cord. She underwent additional open thyroid biopsy and received a nasogastric tube for feeding and was discharged after one week. The open biopsy provided sufficient pathological samples to confirm clinical suspicions, and the patient was diagnosed with at least T4aN1b (stage IVA) anaplastic thyroid carcinoma. At this time, immunohistochemistry (IHC) testing was negative for *BRAF* mutation, and molecular testing of a targeted thyroid malignancy panel via next-generation sequencing (NGS) was pending.

Initial-staging CT thorax revealed multiple bilateral lung nodules, with the largest measuring 11 mm in the left upper lobe, and prominent mediastinal lymphadenopathy, favouring lung metastasis. Further clinical evaluation and re-review of her initial CT neck showed that the patient had significant lymph node metastases in the neck, including retropharyngeal and parapharyngeal lymph nodes with extranodal extension. As a result, the staging was considered T4N2cM1 (stage IVC). A multidisciplinary tumour board recommended concurrent chemoradiotherapy with consideration for immunotherapy. PET scan could not be completed prior to therapy initiation and would not have changed management. The patient was readmitted 7 weeks post-presentation to facilitate a gastrostomy tube in replacement of her nasogastric tube for feeding. Urgent chemoradiation was planned while *BRAF* NGS was pending.

While readmitted, the patient began palliative radiotherapy (66 Gray in 30 fractions), alongside chemotherapy with intravenous carboplatin (204 mg) and paclitaxel (131 mg) every 28 days. However, within 48 h of starting treatment, the patient experienced upper airway obstruction. She was intubated and transferred to intensive care, and further chemotherapy and radiation were discontinued. Several discussions were held regarding goals of care, as the risks of surgery for resection of the mass or for tracheostomy were considered to greatly outweigh the benefits. The patient opted to pursue medical assistance in dying (MAiD).

But surprisingly, the evening before she was set to undergo MAiD, NGS results confirmed the presence of a *^V600E^BRAF* mutation, indicating the potential utility of targeted therapy. This was discussed with the patient and instead of pursuing MAiD, the patient opted for treatment, self-paying for tyrosine kinase inhibitor (TKI) therapy with dabrafenib (75 mg oral twice daily) and trametinib (2 mg oral daily) due to lack of public funding. The response to dabrafenib and trametinib was significant. Follow-up CT of the neck 2.5 months post-presentation demonstrated a partial response, with shrinkage of the thyroid mass and reduction in lymphadenopathy (Figure 1C,G). Her dysphagia and neck stiffness improved, and she was eventually able to be transitioned out of intensive care and was discharged 3.5 months post-presentation with a tracheostomy for airway protection.

Overall, she tolerated targeted therapy well. At approximately 3 months post-presentation and 1 month on dabrafenib-trametinib, she developed grade 3 elevation in alanine aminotransferase and grade 2 elevation in aspartate aminotransferase. At 5 months post-diagnosis and 3 months on dabrafenib-trametinib, she experienced a persistent fever and was admitted for one week. During each side effect, targeted therapy was held for nearly a week, and the patient resumed treatment on symptom improvement. While the patient self-paid for the first cycle of treatment, it was subsequently covered by a compassionate access programme from the manufacturer.

Despite initial tumour shrinkage, she developed a growing neck mass, new hemoptysis, and worsening dysphagia. Repeat CT imaging at 6 months post-presentation revealed disease progression, with increase in size of the thyroid mass and adjacent lymph nodes (Figure 1D,H). The patient discontinued dabrafenib-trametinib and underwent a course of palliative radiation (25 Gray in 5 fractions). She was interested in pursuing second-line systemic treatment and had a PD-L1 combined positivity score greater than 20. The decision was, therefore, made to start on pembrolizumab at 7 months post-presentation; lenvatinib was not utilized due to concerns about fistulization and bleeding risk from vessel encasement.

**Figure 2:**
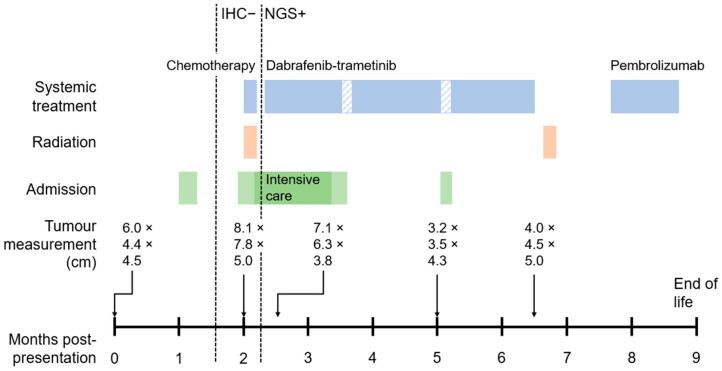
A timeline summary of case details, including the timing of molecular testing results, treatments, admissions, and measurements of the primary thyroid tumour on various computed tomography (CT) images. The patient passed away at 9 months post-presentation. Blue rectangles indicate time on systemic treatment, with white-striped areas indicating treatment holds. Orange rectangles indicate time receiving radiation therapy. Green rectangles indicate time admitted to hospital, with darker green rectangle indicating time in intensive care.

Approximately 1 month after starting pembrolizumab, her condition notably worsened, with increased neck swelling, fatigue, new axillary lymph nodes, and a significant reduction in functional status. Due to clinical deterioration, the patient opted to discontinue pembrolizumab, and she passed away 9 months post-presentation via MAiD. A summary of the case is found in Figure 2.

## 3. Discussion

This case highlights the complexities and potential benefits of targeted therapy in managing *BRAF*-mutated anaplastic thyroid cancer. The patient’s treatment journey provides valuable insights into the investigations and management of this aggressive thyroid cancer.

A critical aspect of this case is discordance in the test results for *^V600E^BRAF* mutations, with an initial falsely negative IHC result and subsequently positive NGS. IHC is utilized as a rapid and cost-effective method for detecting specific mutations [9]. However, it may yield false-negative results due to sample quality, handling, and tumour characteristics [10,11]. The reported sensitivity and the specificity of IHC in detecting *^V600E^BRAF* mutations in ATC patients vary, with sensitivities of 78.9% to 100% and specificities between 69.7% and 95% [11,12,13]. In contrast, NGS provides a comprehensive and accurate analysis of genetic mutations, typically run with multi-gene panels tailored to specific tumour types [14]. A review of *BRAF* mutation detection methods in various non-thyroid carcinomas indicated NGS had a sensitivity of 98.6% and specificity of 100% [15]. However, NGS requires longer turnaround times than IHC [15]. In this case, IHC returned the results 14 days after the patient’s open thyroid biopsy, whereas NGS took an additional 22 days to receive. This time can vary depending on the laboratory, geographical area, and several other factors.

Clinical decision-making must incorporate currently available information on patient and tumour characteristics. While some decisions may be delayed awaiting further information, this is often not possible in rapidly progressive malignancies such as ATC due to evolving functional status. In the 22 days between the negative IHC and positive NGS results, this patient rapidly deteriorated, necessitating intubation and admission to intensive care. Several goals of care discussions were conducted while the tumour was considered *BRAF-*negative. As no available treatments could provide rapid response, and surgery was not possible, the medical team advocated for comfort-focused care and the patient decided to pursue MAiD. Once NGS showed a *^V600E^BRAF* mutation, a rapidly acting treatment option became available, and this decision was re-evaluated. Targeted therapy with dabrafenib and trametinib provided a clinically meaningful extension of her life by nearly 7 months. This scenario highlights that the time to test results can be crucial for decision-making, and delays can have significant implications for treatment in rapidly evolving cancers such as ATC.

Recently, the Idylla™ platform was introduced in Southwestern Ontario to accelerate molecular diagnostics. This fully automated, cartridge-based real-time polymerase chain reaction system detects *^V600E^BRAF* mutations directly from formalin-fixed paraffin-embedded tissue [16,17]. Compared to NGS, Idylla™ offers same-day turnaround times, and has near 100% concordance rate with NGS and other *^V600^BRAF* detection methods in various cancer pathologies [18,19,20]. Sensitivities and specificities are listed in Table 1. The manufacturer cautions that specimen cellularity should be at least 50% for reliable results in its *^V600E^BRAF* testing, potentially limiting utility in FNA-based biopsies, though third-party studies suggest it may remain reliable [21]. In rapidly progressive malignancies such as ATC, where therapeutic decisions must be made within narrow clinical windows, the availability of rapid and reliable molecular testing such as Idylla™ enables earlier initiation of targeted therapies. Liquid biopsy testing, such as circulating tumour DNA, has also shown clinical utility in ATC for identifying driver mutations and potentially tracking disease response [22,23]. Such promising tools can provide profound impacts on patients’ disease trajectory in the future.

Although faster identification of actionable mutations in cancer has proven beneficial for the initiation of specific targeted therapies, access remains an issue. In Canada’s single-payer public health system, some medications may be approved by Health Canada but not approved for public funding. Alternative mechanisms for access include patient support programmes, corporate compassionate access, public exceptional access programmes, private insurance, or self-pay. Pharmaceutical companies may provide formal patient support programmes as a bridge to public funding, which are often time-limited [24]. Companies may rarely provide compassionate access outside approved indications at their discretion; this requires additional time, provider knowledge, and advocacy [25]. Public exceptional access programmes vary province to province, which may create cross-country inequities [24]. Self-pay may occasionally be the only option due to delays, rejections, or lack of formal programmes. This can represent a substantial financial burden: in this case, the patient self-paid approximately CAD 20,000 for the first 28-day cycle of dabrafenib and trametinib [26]. When timely access to life-saving therapy may depend on personal financial capacity, this raises concerns about equitable access [27].

In this patient, dabrafenib and trametinib facilitated significant tumour shrinkage and symptom relief. Her progression-free survival (PFS) with dabrafenib and trametinib was approximately 4.25 months, similar to the median PFS of 4.0 months reported in previous studies [7,28]. Despite initial success with targeted therapy, the patient’s disease ultimately progressed symptomatically and on imaging, necessitating change in treatment. Pembrolizumab was subsequently introduced as second-line therapy, with the duration of response of roughly 4 weeks. Prior reports indicate short median durations of response for PD-L1 monotherapy, though some patients exhibit sustained response [29,30]. While this patient was not considered for combination therapy due to risk of bleed and fistula, lenvatinib-pembrolizumab has shown promise in reviews and small-scale studies [31,32]. Future research is needed to verify optimal treatment options.

The management of this case involved multidisciplinary care, including surgical biopsy, radiation therapy, and systemic treatments. Each modality played a role in managing symptoms and attempting to control the disease, and multimodal therapy is therefore encouraged in guidelines [33]. While it is difficult to quantify the individual benefit each treatment had in this case, together they played a role in management.

## 4. Conclusions

In summary, this case with discordant IHC and NGS test results highlights the impact of molecular testing logistics on clinical decision-making and treatment course in a rapidly progressive malignancy. Rapid and accurate molecular testing is essential to facilitate informed treatment discussions, and earlier results provide additional time to access non-publicly funded therapies that may extend survival and improve quality of life. This patient’s journey emphasizes the need for continued research and innovation in ATC treatment to provide hope and improved outcomes for future patients.

## Figures and Tables

**Table 1:** Summary of sensitivity and specificity rates for various methods of testing for V600EBRAF mutations.

Test	Sensitivity	Specificity
Immunohistochemistry [11,12,13]	78.9% to 100%	69.7% to 95%
Next generation sequencing [15]	98.6%	100%
Rapid real-time polymerase chain reaction (Idylla™) [19]	100%	100%

## Data Availability

The original contributions presented in this study are included in the article. Further inquiries can be directed to the corresponding author.

## Abbreviations

The following abbreviations are used in this manuscript:

ATC	Anaplastic thyroid cancer
CT	Computed tomography
FNA	Fine needle aspiration
IHC	Immunohistochemistry
MAiD	Medical assistance in dying
NGS	Next-generation sequencing
PD-L1	Programmed death ligand 1
PFS	Progression-free survival
TKI	Tyrosine kinase inhibitor
